# Transitioning patients with juvenile idiopathic arthritis to adult care: the Nordic experience

**DOI:** 10.1186/s12969-022-00742-2

**Published:** 2022-10-01

**Authors:** Katriina Mikola, Katariina Rebane, Ellen Dalen Arnstad, Lillemor Berntson, Anders Fasth, Mia Glerup, Troels Herlin, Hannu Kautiainen, Susan Nielsen, Ellen Nordal, Suvi Peltoniemi, Marite Rygg, Veronika Rypdal, Marek Zak, Kristiina Aalto

**Affiliations:** 1 New Children’s Hospital, Pediatric Research Center, University of Helsinki and Helsinki University Hospital, Stenbackinkatu 9, 00290 Helsinki, Finland; 2 Department of Pediatrics, Levanger Hospital, Nord-Trøndelag Hospital Trust, Levanger, Norway; 3 Department of Clinical and Molecular Medicine, NTNU - Norwegian University of Science and Technology, Trondheim, Norway; 4 Department of Women’s and Children’s Health, Uppsala University, Uppsala, Sweden; 5 Department of Pediatrics, Institute of Clinical Sciences, Sahlgrenska Academy, University of Gothenburg, Gothenburg, Sweden; 6 Department of Pediatrics, Aarhus University Hospital, Aarhus, Denmark; 7 Kuopio University Hospital, Primary Health Care Unit Kuopio, Pohjois-Savo, Finland; 8 Folkhälsan Research Center, Helsinki, Finland; 9 Department of Pediatrics, Rigshospitalet Copenhagen University Hospital, Copenhagen, Denmark; 10 Department of Pediatrics, University Hospital of North Norway and Pediatric Research Group, Tromsø, Norway; 11 Department of Clinical Medicine, UIT the Arctic University of Norway, Tromsø, Norway; 12 Helsinki University Central Hospital, HUS Inflammation Center, Rheumatology and University of Helsinki, Helsinki, Finland; 13 Department of Pediatrics, St. Olavs University Hospital, Trondheim, Norway

**Keywords:** Juvenile idiopathic arthritis, Uveitis, Transition, Disease activity, Remission, Follow-up study, Multicentre study

## Abstract

**Background:**

With juvenile idiopathic arthritis (JIA), there are several protocols and practices used worldwide for the transition from paediatric to adult care. In this study, we examined the transferral rates and disease activity after transition, as well as the disease- and health-related outcomes. We also introduce the transition practices employed in the Nordic countries.

**Methods:**

The study population comprised 408 participants with a disease onset from 1997 to 2000 who attended an 18-year follow-up visit in this population-based Nordic JIA cohort study. The patients were retrospectively divided into three subgroups: Patients transferred directly from paediatric care to adult rheumatology care, patients referred there later, and patients never transferred during the 18-year follow-up period.

**Results:**

One hundred and sixty-three (40%) JIA patients had been directly transferred to an adult clinic. The cumulative transition rate was 52%, but there were significant differences between the participating centres. Fifty patients had later been referred to an adult clinic. Among the 195 patients who had never been transferred, 39% were found to have disease activity at the study visit.

**Conclusion:**

This study highlights the need to reconsider transition practices to avoid our undesirable finding of patients with disease activity in JIA, but no appropriate health care follow-up.

## Background

Juvenile idiopathic arthritis (JIA) is a group of diseases beginning before the age of 16 years [1]. Although the use of biological medications has changed the disease outcome [2], half of patients with JIA still have an active disease at the time of their transfer to adult care [3, 4]. Transferring young people with a chronic disease to adult care is a vital process to guarantee the continuity of disease management. The human brain undergoes many physiological changes during the adolescent period [5] and these may create specific challenges to the maintenance of chronic disease control in the time period of an ongoing transition process to adult care.

There are several different practices and protocols for transferring to adult care in paediatric rheumatology [6, 7]. Recently, a European League Against Rheumatism (EULAR) and Paediatric Rheumatology European Society (PReS) recommendation about transition in JIA was published [8], but no congruent program accepted worldwide is in use. Special transition programmes have been shown to improve patients’ outcomes, i.e. reduce dropouts from care and enhance patients’ self-efficacy skills [9]. The transition process is more than the actual transferal to an adult clinic; it should begin several years in advance and include individual planning both in the pediatric and in the adult clinic, as well as their cooperation. Timing should be based on individual divergences in physical, emotional and social maturing [6, 10].

We have previously studied the transitions of Finnish patients with JIA [11] and found that, although 40% of the patients were transferred directly from the paediatric unit into adult care, up to 72% of the patients ended up there during the 17-year follow-up. The observed high rate of cumulative referral in this study indicates the necessity for critical inspection of transition practices in JIA.

In the present study, we retrospectively collected information on referral to an adult rheumatology clinic from a prospective, longitudinal Nordic JIA cohort study. Furthermore, we examined the disease activity after the transition, as well as disease- and health-related characteristics. Our main focus is on transition rate, i.e., how many patients are transferred around the age of 16–18. In addition, we present the cumulative transition rate and estimate the used transition practices in that perspective. A synopsis of transition practices in participating Nordic sites is given.

## Methods

In this Nordic JIA cohort follow-up study, all newly diagnosed consecutive JIA patients from the specified geographical areas of Denmark, Finland, Norway and Sweden were selected during the inclusion period, with disease onset from January 1, 1997 to June 30, 2000 [12]. This prospective cohort study was designed as a population-based study. To reach all eligible patients, letters were sent to all primary health care centres and orthopaedic, rheumatology and paediatric clinics in the catchment areas [13]. The patients were followed at study visits eight and 18 years after the disease onset, as previously described in more detail [13]. The patient population has previously been described [14]. All original patients (*n* = 510) were invited to participate in the 18-year follow-up study. For those who could not participate in the hospital visit, a standardised telephone interview was carried out, and they filled out electronic patient questionnaires as well [14]. The 18-year follow-up visit included a joint and ophthalmologic examination, the collection of clinical data and updating of medical histories. The preceding disease status within the period of the 8–18-year study visits was assessed. JIA categories were classified according to the International League of Associations for Rheumatology (ILAR) criteria [15]. There were no exclusion criteria, except those 26 patients who were still attending the paediatric unit.

Of the 434 individuals participating in the 18-year follow-up, 408 were included in this transition study. Those 76 individuals lost from follow-up showed no significant differences in basic characteristics compared to those included in the study [14]. In the 18-year follow-up study, we retrospectively gathered information on the previous transition process. The patients were divided into three groups: ‘directly transferred’, ‘later referred’ and ‘not transferred’. The patients in the latter two groups had not been directly transferred to adult care when leaving paediatric clinic. However, the patients in the ‘later referred’ group had been referred at some point during the follow-up period, due to suspicion of the disease activity. Adult rheumatologists have not seen the patients in the ‘not transferred’ group at any point during this study’s follow-up period.

Remission was defined according to the preliminary Wallace criteria, in which the criterion needed for inactive disease are given and remission is divided into remission on or off medication [16]. The Juvenile Arthritis Damage Index (JADI) was used to assess articular and extra-articular damage [17]. JADI-A scores articular damages, i.e. permanent contractures or past surgical interventions, and JADI-E scores extra-articular damage — for example, complications related to uveitis [17]. Information about the past transition status was collected, i.e., if patients were directly transferred to an adult rheumatology clinic, referred later or never, or if they were still visiting the paediatric rheumatologist. Patient reported outcomes were measured using the Health Assessment Questionnaire (HAQ) and visual analogue scales (VASs) for pain, global assessment of well-being and fatigue [18]. Disease Activity Scores (DAS28) and physician-reported global assessment of disease activity on a 21-numbered VAS were collected [19]. Health-related quality of life was assessed using SF-36 questionnaire [20, 21] with value 50 defined as a norm and lower values indicating reduced physical or mental health. Patients also reported the frequency of physical exercise, their use of physiotherapy and their socioeconomic status.

### Description of transition practices in the Nordic countries

The transition practices used in the participating areas during the follow-up period are shown in Table 1. The Nordic countries and their geographical areas and sites participating in this study were explained more thoroughly in the previous article, which has a map of the geographic areas [12]. At all participating sites, the transition practices underwent changes during this study period. At most sites, the transition process started well before the actual referral to adult care took place. There were variations in whether the adolescents were seen alone during the consultation at outpatient clinics; this occurred at half of the sites. Finland is the only country where the transition age is 16 years, in the other Nordic countries the transition age to adult care is typically 18 years. Active disease or ongoing medication was usually essential for transition. There was not necessarily a specific transition clinic at every adult site, and the policy of collaboration between the adult and paediatric sites differed; for example, regular meetings with the paediatric and adult sites were not arranged at all sites. An open website (Healthvillage.fi) produced by university hospitals in Finland, where adolescents can find general information about JIA as well as information about transitioning and adult clinics, was created during this study period.

**Table 1:** Transition practices in participating Nordic study sites

Transition practices	DEN/ Copenhagen	DEN/ Århus	FIN Helsinki	NOR/ Tromsø	NOR/ Trondheim	SWE/ Multiple sites
1. A multidisciplinary team with appropriate education is responsible for the treatment and care of young people (YP) with juvenile idiopathic arthritis (JIA) during the transition process YES/NO	YES	YES	YES	YES	YES	YES
a. paediatric rheumatologist	YES	YES	YES	YES	YES	YES
b. paediatric rheumatologist nurse	YES	YES	YES	YES	YES	YES
2. The transition process starts well in advance in early adolescence (between 11 and 14 years, or immediately if the diagnosis is made later) YES/NO/ Varying	NO	YES	YES	YES	YES	Varying
3. Adolescents are first seen alone at the appointment and the parents join the visit later YES/NO/ Varying	NO	YES	YES	Varying	NO	Varying
4. The average age at transition YEAR	18	18	16	18	18	18
5. There is a transition program with special categories or a checklist to complete before the transferal to an adult clinic YES/NO	NO	YES	YES	YES	NO	NO
6. YP with JIA regularly fill in health-related questionnaires and estimate their VAS-values YES/NO	YES	YES	YES	YES	YES	YES
7. There are physiotherapist and/or psychologist and/or occupational therapist and/or rehabilitation counsellor services before the transferal YES/NO						
a. at least one of the above-mentioned services is mandatory for all	NO	YES	NO	YES	YES	NO
b. if needed	YES	YES	YES	YES	YES	YES
8. Criteria for transition YES/NO						
a. all patients are transferred	NO	NO	NO	NO	NO	NO
b. active disease/ongoing medication is needed	YES	YES	YES	YES	NO	YES
9. There is a specific transition clinic at the adult rheumatology clinic with engagement in adolescent rheumatology YES/NO	NO	YES	YES	No	YES	NO
10. There is direct communication with the paediatric and adult team before the actual transferal YES/NO						
a. for every YP with JIA	NO	YES	NO	NO	NO	NO
b. for complex cases	YES	YES	YES	YES	YES	YES
11. YP with JIA visit the adult clinic in advance YES/NO/IF NEEDED	NO	YES	IF NEEDED	YES	YES	IF NEEDED
12. There are regular meetings with the paediatric and adult teams to evaluate the transition practise YES/NO/varying	YES	YES	YES	NO	YES	Varying
13. There is a freely accessible electronic webpage about transition YES/NO	NO	NO	YES	NO	NO	NO

*DEN* Denmark, *FIN* Finland, *NOR* Norway, *SWE* Sweden

The information on transition practices selected for Table 1 is based on the EULAR/PReS recommendations for transition [8] and was collected from the participating study sites. The principles of the healthcare systems in the Nordic countries are quite parallel: The public welfare sector is funded by the taxation system and is basically free or low-cost for citizens at the point of delivery (www.norden.org), and the social welfare system may provide additional financial support if necessary. The public healthcare system is open to all inhabitants without restrictions, and the most costs of medications, such as expensive biological drugs, are compensated by the state. Therefore, the transition procedure with possible ongoing medications requires no extra financial investment from the patients.

### Analyses

The descriptive statistics were presented as means with SDs or as counts with percentages. Statistical comparisons between the three transferred groups were performed using analysis of variance (ANOVA) and a chi-square test. The relationships between the transfer groups and the remission of DAS28 values were evaluated using two-way ANOVA. The models included main effects (transferred groups and remission) and interaction effects between them. In the case of the violation of the assumptions (e.g., non-normality) for continuous variables, a bootstrap-type method or Monte Carlo *p*-values (small number of observations) for categorical variables were used. Hommel’s adjustment was applied to correct levels of significance for multiple testing (post hoc testing) if appropriate. The normality of variables was evaluated graphically and by the Shapiro–Wilk test. The Stata 17.0 (StataCorp LP; College Station, Texas, TX, USA) statistical package was used for the analysis.

## Results

The clinical characteristics are shown in Table 2. Significant differences were found between the transfer groups and in the age at onset of the disease as well as the category of the disease. Seventy-nine patients (48%) of those who were directly transferred had seronegative polyarthritis or extended oligoarthritis. Persistent oligoarthritis was the main category in those who were not transferred, and they were also the youngest at the onset of JIA.

**Table 2:** Clinical characteristics of the study population at the 18-year study visit

	Directly transferred (D) (*n* = 163)	Later referred (L) (*n* = 50)	Not transferred (N) (*n* =195)	*P*-value [multiple comparison] *
Female, n (%)	116 (71)	39 (78)	124 (64)	0.091
Age at onset, years, mean (SD)	7.3 (4.2)	7.7 (3.8)	6.3 (4.1)	0.029 [D/N]
Age at follow-up, years, mean (SD)	25 (4)	25 (4)	24 (4)	0.30
Body mass index, kg/m2, mean (SD)	24.4 (5.0)	23.5 (4.3)	23.8 (4.7)	0.41
JIA category^a^ n (%)				< 0.001 [D/N, L/N]
Systemic onset	3 (2)	0 (0)	11 (6)	
Polyarthritis, RF+	3 (2)	0 (0)	3 (2)	
Polyarthritis, RF-	37 (23)	8 (16)	18 (9)	
Juvenile psoriatic	12 (7)	4 (8)	11 (6)	
Enthesitis-related	24 (15)	6 (12)	13 (7)	
Undifferentiated	25 (15)	7 (14)	29 (15)	
Persistent oligoarthritis	17 (10)	12 (24)	86 (44)	
Extended oligoarthritis	42 (26)	13 (26)	24 (12)	

*SD* Standard deviation, *JIA* Juvenile idiopathic arthritis, *RF* Rheumatoid factor

*Hommel’s multiple comparison procedure was used to correct significance levels for post hoc testing (*p* < 0.05)

^a^According to the ILAR classification criteria [16]

Disease and health-related outcomes and medications used at the study visit are shown in Table 3. One hundred sixty-three (40%) of the 408 JIA patients were directly transferred to an adult clinic. Fifty patients were referred later, and 195 had not been transferred during the 18-year follow-up.

**Table 3:** Disease and health-related outcomes and medications used at the study visit

	Directly transferred (D) (*n* = 163)	Later referred (L) (*n* = 50)	Not transferred (N) (*n* = 195)	*P*-value [multiple comparison] *
HAQ, mean (SD)	0.32 (0.52)	0.13 (0.32)	0.05 (0.27)	< 0.001 [D/L, D/N]
Patients with active joints, n (%)	45 (28)	9 (18)	8 (4)	< 0.001[D/L, D/N, L/N]
Number of cumulative active joints, median (IQR)	15.2 (11.8)	8.8 (8.2)	5.3 (5.6)	< 0.001 [D/L, D/N, L/N]
DAS28, mean (SD)	2.21 (1.31)	1.79 (1.00)	1.29 (0.75)	< 0.001 [D/L, D/N, L/N]
VAS 0–100 mean (SD)				
Pain	27 (26)	21 (26)	9 (16)	< 0.001 [D/N, LN]
Physicians’ global	13 (20)	8 (16)	2 (8)	< 0.001 [D/N, L/N]
Patients’ global	27 (29)	18 (23)	8 (16)	< 0.001 [D/L, D/N, L/N]
Fatigue	43 (28)	36 (27)	35 (27)	0.045 [D/N]
JADI-A, n (%)				< 0.001 [D/L, D/N, L/N]
0	115 (71)	43 (86)	187 (96)	
1	19 (12)	4 (8)	5 (3)	
2–4	21 (13)	2 (4)	3 (2)	
≥ 5	8 (5)	1 (2)	0 (0)	
CAM, n (%)	21 (13)	6 (12)	22 (11)	0.90
Medication, n (%)	95 (58)	14 (28)	0 (0)	< 0.001 [D/N, L/N]
Synthetic DMARDs, n	62	11	0	
Biologic DMARDs, n	65	4	0	
Systemic steroids, n	8	2	0	
SF-36, mean (SD):				
Physical component score	47.8 (10.8)	49.5 (10.5)	55.5 (6.5)	< 0.001 [D/N, L/N]
Mental component score	47.7 (12.7)	51.2 (8.6)	49.8 (11.0)	0.12
Socioeconomics n (%)				0.089
Student	71 (45)	17 (35)	85 (44)	
Working	68 (43)	28 (58)	98 (51)	
Unemployed	5 (3)	2 (4)	5 (3)	
Disability pension	14 (9)	1 (2)	5 (3)	
JIA-related	6 (4)	0 (0)	0 (0)	
Other reasons	8 (5)	1 (2)	5 (3)	
Physical exercise				0.68
< once a week	24 (17)	5 (13)	19 (12)	
1–3 times a week	89 (61)	26 (67)	102 (62)	
4–7 times a week	32 (22)	8 (21)	43 (26)	
Physiotherapy, ongoing, n (%)	17 (11)	4 (10)	6 (4)	0.030 [D/N]
Inactive disease^a^ off medication between 8 and 18 year study visits, n (%)	10 (6)	8 (16)	146 (75)	< 0.001 [D/L, D/N, L/N]
JIA-U ever, n (%)	41 (25)	15 (30)	18 (9)	< 0.001 [D/N, L/N]

D Directly transferred, L Later referred, N Not transferred

* Hommel’s multiple comparison procedure was used to correct significance levels for post hoc testing (*p* < 0.05)

^a^According to the Wallace preliminary criteria [16]

*SD* Standard deviation, *IQR* Interquartile Range, *HAQ* Health Assessment Questionnaire, *DAS28* Disease activity score, *VAS* Visual Analogue Scale, *JADI-A* The Juvenile Arthritis Damage Index assessment of articular damage, *JIA-U* Juvenile idiopathic arthritis-related uveitis, *CAM* Complementary and alternative medicine, *DMARDs* Disease-modifying anti-rheumatic drugs, *SF-36* 36-item Short Form Health Survey, *JIA* Juvenile idiopathic arthritis

In the ‘not transferred’ group, 39% (*n* = 76) of the patients were found not to be in remission at the study visit according to the Wallace criteria, although only eight patients had active joints at the study visit. However, 75% (*n* = 146) of patients in this group had had remission off medication at some point during the preceding 10 years compared to 6% (*n* = 10) in the ‘directly transferred group’. The cumulative number of active joints was also higher in the ‘directly transferred’ group. At the study visit, 74% (*n* = 121) of directly transferred patients were still attending the rheumatology clinic, compared to 48% (*n* = 24) in the ‘later referred’ group.

The prevalence of JIA-related uveitis (JIA-U) was lowest in the ‘not transferred’ group; only 9 % (*n* = 18) had a history of uveitis. Twenty-five percent (*n* = 41) of the directly transferred patients had had uveitis, but so did also 30% (*n* = 15) of those who were later referred.

There were no differences in socioeconomic status between the three groups. The physical component score in SF36 was lower, indicating reduced physical health in the ‘directly transferred’ and ‘later referred’ groups compared to the ‘not transferred’ group.

At the study visit, there was no statistically significant difference between the use of CAMs and the remission rate or the DAS28 value.

**Fig. 1:**
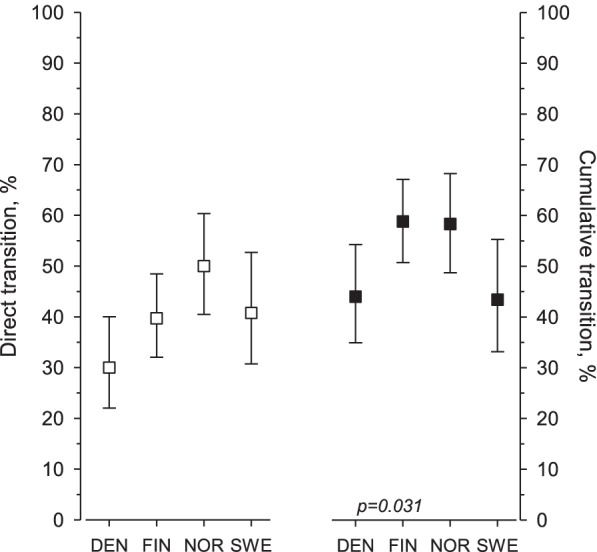
The direct and cumulative transition rates during the total follow-up time to an adult clinic in the prospectively followed Nordic Juvenile Idiopathic study cohort. The white squares show the percentage of patients who were directly transferred to an adult site in each country, and the black squares show the cumulative transition rate to an adult site during the study’s 18-years follow-up period. The results in each participating country are pooled together due to the low number of patients at different sites which are shown in Table 1. DEN = Denmark, FIN = Finland, NOR = Norway, SWE = Sweden

The proportion of directly transferred patients was 40% (Fig. 1) and the cumulative transition rate during the total follow-up time was 52%. There was a statistically significant difference in the total cumulative rate between the Nordic sites; in Finland and Norway, the rate was highest.

**Fig. 2:**
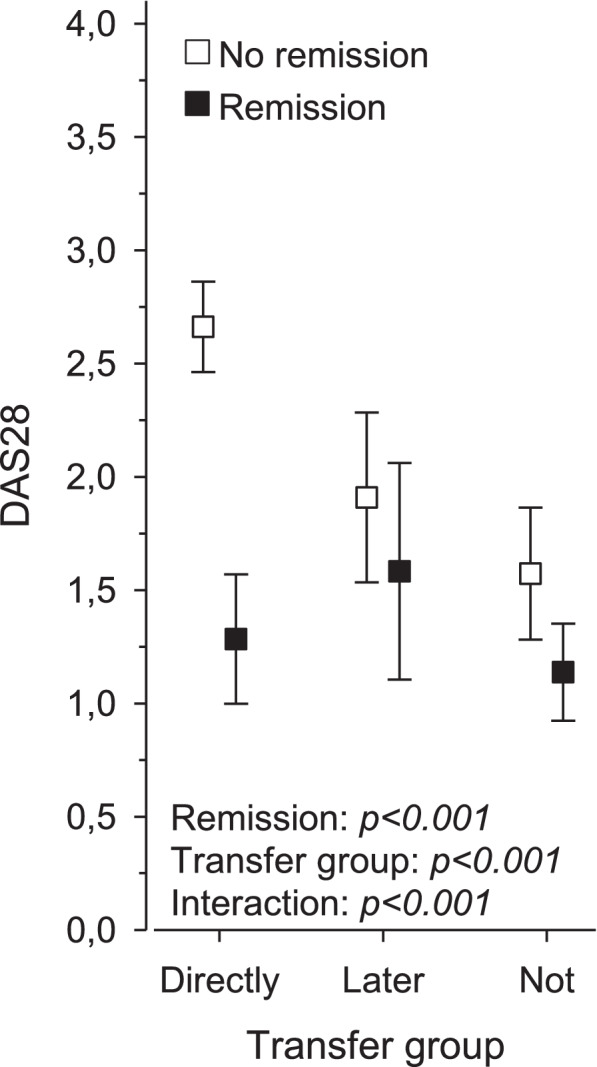
Remission status at the 18-year follow-up and DAS28 scores in three different transfer groups in the Nordic juvenile idiopathic arthritis cohort study. The white squares represent patients whose disease was not in remission and the black squares represent patients who were in remission at the 18 year-follow-up visit. The relation between remission status and DAS28 score as well as their interactions, are shown in three different transition groups. DAS28 = Disease activity score

Figure 2 shows the different transfer groups according to their remission status at the 18-year follow-up and their relationship to the disease activity (DAS28) values. Those who were directly transferred and had an active disease at the 18-year follow-up also had the highest DAS28 values. The main effects of the remission status (*p* < 0.001), transfer group (*p* < 0.001) and their interaction (*p* < 0.001) were statistically significant. The directly transferred group had the highest difference in DAS28 related to remission status.

## Discussion

The main finding in our study is that a relatively high proportion of patients who were not transferred to an adult clinic had an active disease at the 18-year follow-up study visit, despite previously being in long-term remission off medication. However, their disease activity was mild and had a minimal impact on their daily lives. On the other hand, those who were directly transferred had probably had a more severe disease course at the time of their transition to the adult clinic, and most of them still had either active disease, or ongoing medication, and regular appointments at the adult rheumatology clinic at their 18-year follow-up study visit. These findings could indicate that the appropriate decision concerning transition was made in the past. However, whether young adults with a history of JIA either do not recognize the joint symptoms or do not have the information needed to reach the health care system for evaluation remains to be considered. Patients with JIA may be habituated to their ongoing pain and discomfort caused by the disease, and these experiences may have given them a tenacious character with a higher threshold to seek medical help. However, we did not investigate these factors in our study. As a conclusion, it can be considered that a different kind of transition system might have nonetheless benefitted these young adults.

In our study, 40% of the patients were directly transferred to an adult clinic. Considering the previous findings in this study group, one might expect the transition rate to be higher when it is noted that 49 and 53% of the patients with JIA were not in remission after 8 years and 18 years from diagnosis, respectively [13, 14]. The cumulative transition rate of just above 50 % in our study could also be considered too low in light of the documented chronic nature of JIA [13, 14, 22, 23].

The ‘directly transferred’ and ‘later referred’ groups were similar in many aspects in our study; patients in those groups were older at the disease onset, thus with a shorter follow-up period in the paediatric ward, and they had more polyarthritis and extended oligoarthritis compared to the ‘not transferred’ group. A more detailed analysis of the ‘later referred’ group could reveal aspects of whether they should have been transferred directly as well. Nevertheless, only half of them were still attending an adult rheumatology clinic, so the symptoms leading to a later referral were supposedly mild. Our deduction is that too many patients were incorrectly assessed outside the transition to adult care, as their disease was activated later, and they needed to be referred to an adult clinic. The worrying finding was the relatively large amount (39%) of patients that had an active disease without an appropriate healthcare contact. These concerns could be avoided by transferring all patients, but in most sites the resource constraints cause some limitations.

A recent article revealed detailed information about uveitis in this Nordic study population [24]. It was discovered that, during the 18-year follow-up, 22% had had uveitis at some point. In our current transition study, a relatively high number of patients with JIA-U at some point of the disease course were not transferred directly to the adult clinic. Chronic uveitis is the most common extra-articular manifestation of JIA [25], and it has been shown to increase the disease burden in young patients with JIA [26]. However, we did not focus more closely to the transition in uveitis in our study and more studies are needed to investigate the need for special transition procedures also in JIA-U.

We observed some differences in the transition practices between the different sites in the Nordic countries participating in this study. However, no study site had a special protocol in the transition process at the beginning of the twenty-first century. Since 2010, most of the sites participating in this study have developed their processes for transition, and this work will undoubtedly create better and more correctly focused transitions. In an article by Conti et al. [7], different kinds of transitional programmes around the world were introduced. They report that actual timing of transition varies; apart from the age (usually 16–18 years, up to 21), it can be linked to a patient’s educational stage or to the level of overall disease and treatment awareness, whereas in some centres, it can be assessed quite individually. The transition age of 16 could be seen as logical and congruent with the present JIA classification criteria [15]. Nevertheless, some unfavourable development, a functional decline in performance concerning relational reasoning for example, has been observed in adolescents [27]. During normal brain development however, there is an increased activation of prefrontal brain regions [28], and consequently, favourable progress towards more goal-directed behaviour with better control of emotions, social functioning and learning and less impulsivity and risk-taking behaviour [29]. These matters most certainly have an influence on adolescents’ competence in disease management. Ongoing development may further reduce the success of a transition and must be considered when establishing the appropriate age for transition.

The patients in the ‘directly transferred’ group were most often still using anti-rheumatic medications at the study visit. This is in line with the transition procedures in the Nordic countries, i.e., transition to an adult clinic commonly involves ongoing medication. In the Finnish study by Nousiainen et al. [30], the use of complementary and alternative medicine (CAM) was found to be relatively frequent among adolescents with JIA: 81% reported occasional use. In our study, CAM usage was not very frequent: around 12% of patients reported it, and there were no differences between the three transition groups. The higher CAM use among patients with JIA could be worrying; it might indicate the risk of neglecting the antirheumatic medication [31]. However, in our study patients with active disease did not use more CAMs compared to others.

In our study, we did not find a difference between the socioeconomic status and the level of physical exercise among the three study groups. The use of physiotherapy was highest among the patients transferred directly and referred later, but altogether, a relatively small number of patients—11 and 10%, respectively—received physiotherapy at the 18-year follow-up. There was a difference in physical functioning between the study groups, indicating lower performance in both transition groups. Nevertheless, the number of patients who exercised regularly was pleasingly high; around two-thirds in all groups did some exercise one to three times a week. There were no differences in mental functioning between the groups in our study. Possible concomitant psychiatric disorders are nevertheless important to take into consideration during the transition phase: patients with JIA and psychiatric diagnoses were found to have a lower quality of life, even when the disease was in remission [32].

The strength of this study is the long-term follow-up of this cohort in collaboration between the participating sites. This is the first study reporting transition practices at multiple sites in Nordic countries. However, further studies are needed to make comprehensive conclusions about the transition in each country. The lack of congruent transition protocols in the Nordic countries makes it challenging to draw definitive conclusions. It is also a limitation that data on transition practices during the study period was collected retrospectively. Further research on those patients not directly transferred might yield valuable information for the future development of transition protocols. The need for further studies concerning transition is obvious, especially among patients with JIA-associated uveitis.

## Conclusion

A considerable number of patients were found to have an active disease at the study visit, yet these patients had no appropriate rheumatological care. Young adults with a history of rheumatic disease require easy and well-timed availability of a competent clinical evaluation if needed, after the regular follow-up during childhood and adolescence has ended. The number of patients who were directly transferred to adult care and the cumulative transition rate in the present study is relatively small taking into consideration that JIA is a chronic disease. The fundamental question is how to choose those patients who need to be transferred to an adult unit when they leave paediatric care.

## Data Availability

Data is available upon reasonable request from Nordic Study Group of Pediatric Rheumatology (NoSPeR).

## Abbreviations

JIA: Juvenile idiopathic arthritis
JIA-U: JIA-related uveitis
EULAR: European League Against Rheumatism
PReS: Paediatric Rheumatology European Society
ILAR: The International League of Associations for Rheumatology
JADI: The Juvenile Arthritis Damage Index
JADI-A: The Juvenile Arthritis Damage Index, articular damage
JADI-E: The Juvenile Arthritis Damage Index, extra-articular damage
HAQ: The Health Assessment Questionnaire
DAS28: Disease Activity Score
SF-36: Short Form Health Survey questionnaire
ANOVA: Analysis of variance
CAM: Complementary and alternative medicine
